# Serological Profile for Major Respiratory Viruses in Unvaccinated Cows from High-Yielding Dairy Herds

**DOI:** 10.3390/ani14091256

**Published:** 2024-04-23

**Authors:** Geovana Depieri Yoshitani, Stefany Lia Oliveira Camilo, Juliana Torres Tomazi Fritzen, Marcos Vinicius Oliveira, Elis Lorenzetti, Julio Augusto Naylor Lisbôa, Alice Fernandes Alfieri, Amauri Alcindo Alfieri

**Affiliations:** 1 Laboratory of Animal Virology, Department of Veterinary Preventive Medicine, Universidade Estadual de Londrina, Londrina 86057-970, PR, Brazil; geovana.depieri@uel.br (G.D.Y.); jufritzen@uel.br (J.T.T.F.); marcos.biom@uel.br (M.V.O.); lorenzettielis@uel.br (E.L.); aalfieri@uel.br (A.F.A.); 2 Departament of Veterinary Clinics, Universidade Estadual de Londrina, Londrina 86057-970, PR, Brazil; stefanyliacamilo@hotmail.com (S.L.O.C.); janlisboa@uel.br (J.A.N.L.); 3 Post Graduate Program in Animal Health and Production, Universidade Pitágoras Unopar Anhanguera, Arapongas 86702-670, PR, Brazil; 4 Multi-User Animal Health Laboratory, Molecular Biology Unit, Department of Veterinary Preventive Medicine, Universidade Estadual de Londrina, Londrina 86057-970, PR, Brazil; 5 National Institute of Science and Technology for Dairy Production Chain (INCT–LEITE), Universidade Estadual de Londrina, Londrina 86057-970, PR, Brazil

**Keywords:** serology, virus neutralization test, bovine alphaherpesvirus 1, bovine viral diarrhea virus, bovine parainfluenza virus 3, bovine respiratory syncytial virus

## Abstract

**Simple Summary:**

Bovine Respiratory Disease (BRD) is a major health issue in dairy farming worldwide. It commonly affects lactating and recently weaned calves, but dairy cows can also be impacted resulting in significant losses to the milk production chain. This research aimed to investigate the serological status of dairy cows for the four main bovine respiratory viruses. This study took place in the central-eastern mesoregion of the state of Paraná, which has the highest milk productivity in Brazil. A total of 497 serum samples were assessed from unvaccinated dairy cows from 39 herds. This study aimed to determine the presence of antibodies to bovine alphaherpesvirus 1 (BoAHV1), bovine viral diarrhea virus (BVDV), bovine parainfluenza virus 3 (BPIV3), and bovine respiratory syncytial virus (BRSV) using virus neutralization tests. The frequencies of BoAHV1, BVDV, BPIV3, and BRSV seropositive cows and herds were 71.4, 56.3, 96.8, and 63.4%, and 79.5, 82.0, 100, and 84.6%, respectively. Rates of more than 50% of cows and herds seropositive for the 4 viruses indicate that these respiratory pathogens are endemic in dairy herds in the region evaluated. In this region for BRD prevention, we recommend implementing cow vaccination programs that provide passive immunity in calves and active immunity in cows.

**Abstract:**

This study aims to determine the serological profile of high-yielding dairy cows for four main viruses (bovine alphaherpesvirus 1 (BoAHV1), bovine viral diarrhea virus (BVDV), bovine parainfluenza virus 3 (BPIV3), and bovine respiratory syncytial virus (BRSV)) related to bovine respiratory disease (BRD) in cattle herds worldwide. In this survey, 497 blood serum samples were collected from non-vaccinated dairy cows without clinical respiratory signs in 39 herds in the central-eastern mesoregion of Paraná State, South Brazil. The presence of neutralizing antibodies was determined by virus neutralization (VN) tests. VN antibodies against BoAHV1, BVDV, BPIV3, and BRSV were detected in 355 (71.4%), 280 (56.3%), 481 (96.8%), and 315 (63.4%) serum samples, respectively. The frequencies of seropositive herds for BoAHV1, BVDV, BPIV3, and BRSV were 79.5 (*n* = 31), 82.0 (*n* = 32), 100 (*n* = 39), and 84.6% (*n* = 33), respectively. The frequencies of seropositive cows varied according to the type of herd management and the number of cows in the herd. The detection of VN antibodies in unvaccinated dairy cattle herds demonstrated the endemic circulation of the four viruses in the herds evaluated. For BRD prevention, it is recommended to implement a vaccination program for cows that provides passive immunity in calves and active immunity in cows.

## 1. Introduction

Brazil has the world’s largest commercial cattle herd, with more than 234 million cattle heads [1]. The country is also the largest milk and milk products producer in Latin America, the second on the American continent, and the sixth in the world [2]. The Brazilian dairy cattle herd has 15.9 million milked cows and produces approximately 35.3 billion liters of milk/year. Dairy farming influences the economic growth of Brazil, contributing USD 13.8 billion by 2021 [1]. The southern region stands out in first place in the ranking of milk productivity per cow and is the second-largest national producer. Paraná is the third-largest dairy herd in Brazil and the second milk producer in the country. The central-eastern mesoregion of Paraná has counties with high milk production and productivity [1].

The intensification of production systems leads to an increase in the occurrence of infectious diseases, which negatively affect livestock development [3]. Bovine respiratory disease (BRD) has a considerable economic impact on cattle production worldwide [4].

BRD complex is multifactorial and multi-etiological. An imbalance between the environment (weather and facilities), hosts (age and immunological profile), and infectious agents such as viruses and bacteria is involved in triggering BRD, especially in dairy cattle herds and in beef cattle feedlots [5]. Cows with BRD exhibit decreased milk production, reduced weight gain, and increased production costs [6]. However, infections are more frequent in suckling and weaned heifer calves with delayed body development [7].

The viral agents most associated with BRD include bovine alphaherpesvirus 1 (BoAHV1), bovine viral diarrhea virus (BVDV), bovine parainfluenza virus 3 (BPIV3), and bovine respiratory syncytial virus (BRSV). *Mannheimia haemolytica*, *Pasteurella multocida*, *Histophilus somni*, and *Mycoplasma bovis* are the bacteria identified with the highest frequencies [7,8]. Viral and bacterial infections can occur as single infections or, more often, as co-infections, influencing the severity of clinical signs [7].

BoAHV1 is a linear double-stranded DNA virus belonging to the *Orthoherpesviridae* family [9]. After initial replication in mucous membranes, the virus ascends to neuronal cells, developing a latent infection, making the animal carriers and potential spreaders of the virus in the herd. In addition to the respiratory system, BoAHV1 can also affect the reproductive and genital systems. BoAHV1 can also interfere with the pathophysiological mechanisms of animals, predisposing them to secondary bacterial infections [10].

BVDV is a single-stranded, positive-sense RNA virus that belongs to the *Flaviviridae* family [9]. In a specific gestational period in which the infection occurs (40 to 125 days of gestation)*,* cows can give birth to persistently infected calves [11]. These animals have great epidemiological importance, as they act as reservoirs and spreaders of viruses in herds. Similarly to BoAHV1, BVDV infection does not affect the respiratory tract alone. Acute BVDV infections range from subclinical in immunocompetent animals to respiratory, reproductive, enteric, and cutaneous diseases. In cattle, BVDV causes a reduction in circulating lymphocytes and lung macrophage function, leading to immunosuppression [12,13].

BPIV3 is a single-stranded positive-sense RNA virus classified in the family *Paramyxoviridae* [9]. Infections in cattle can be subclinical or cause mild respiratory clinical signs. As a result of immunosuppression, BPIV3 may act in the predisposition to secondary bacterial pneumonia [14].

BRSV is a single-stranded, positive-sense RNA virus belonging to the *Pneumoviridae* family [9]. Clinical signs of infection range from mild upper respiratory signs to pulmonary infection, followed by pneumonia. BRSV also compromises the defense mechanisms of the respiratory tract, predisposing animals to secondary bacterial infections [15].

In Brazil, several seroepidemiological surveys with an approach to reproductive diseases in beef and dairy cattle have evaluated the serological profile of cows for BoAHV1 and BVDV infections [16,17,18]. Regardless of the goal of production (beef or dairy), management practices (extensive, semi-intensive, or intensive), genetics (*Bos taurus taurus* or *Bos taurus indicus*), and the Brazilian geographic region, among other characteristics, studies shows that both infections are endemic in Brazilian bovine livestock.

Seroepidemiological studies on BPIV3 and BRSV infections, which are mostly restricted to the bovine respiratory tract, are less frequent than those on BoAHV1 and BVDV. These studies were often conducted on animals of age groups that are more susceptible to respiratory infections, such as young animals, including suckling and newly weaned calves [19,20,21,22,23]. However, worldwide, including in Brazilian livestock, the seroepidemiological profile of cattle herds shows that BRSV and BPIV3 infections are frequent [15,24].

Passive immunity plays a vital role in protecting suckling calves. To analyze the risk of infection in both suckling and recently weaned calves, it is important to know the seroepidemiological profile of cows from dairy cattle herds that have not been vaccinated for viruses known to cause BRD. In cattle herds with a low frequency of seropositive cows, either through natural infection or vaccination, the risk of BRD in calves is higher. Therefore, it is necessary to adopt risk mitigation measures in such cases. This study aimed to evaluate the seroepidemiological profile of unvaccinated cows for viral agents causing BRD in high-yield dairy farms located in the central-eastern mesoregion of the state of Paraná.

## 2. Materials and Methods

### 2.1. Sampling

To determine the required number of dairy herds and blood serum samples for evaluation, we used EpiInfo 7.4.2.0 from the Centers for Disease Control and Prevention in Atlanta, GA, USA. We obtained the total number of milked cows (approximately 63,500) in the counties of Arapoti, Castro, Carambeí, Palmeira, and Ponta Grossa from the 2017 Census of Agriculture [25]. Since there were no available data on the seropositive cows for the four respiratory viruses in the central-eastern mesoregion (24°19′26″ S and 50°36′57″ W) of Parana State, we estimated the prevalence as 50% and the design effect as 1.5, with a confidence interval of 95% [26]. To ensure statistically significant conclusions and a safety margin, we collected blood serum samples from 497 cows aged over 2 years without any clinical signs of BRD. Thirty-nine unvaccinated high-yielding dairy cattle herds were selected based on convenience. These herds had an average milk production of 32.6 (25.5–40.1) liters per cow per day, representing approximately 3% of the dairy herds in the 5 counties. The sample size in each county was determined using the proportional allocation method [26], which accounted for approximately 1% of the total number of milked cows. The number of cows studied per herd varied by county and had to be restricted based on the number of farms from which we could collect samples for our analysis [27]. The herds varied significantly in size, ranging from 33 to 3773 cows. We assessed dairy herds with extensive, semi-intensive, and intensive cow management. Serum samples were collected between October 2019 and January 2020 and stored at −20 °C in the bank of serum of the Laboratory of Animal Virology at the Universidade Estadual de Londrina (UEL). This study was approved by the Ethics Committee on Animal Use (CEUA/UEL protocol no. 1835.2019.45).

### 2.2. Virus Neutralization Test

The virus neutralization (VN) test for BoAHV1 and BVDV was performed according to the Manual of Diagnostic Tests and Vaccines for Terrestrial Animals of the World Organisation for Animal Health (WOAH) [28] and for BRSV and BPIV3 according to Affonso et al. [29] and Okur-Gumusova et al. [30], respectively. Serum samples were initially inactivated at 56 °C for 30 min and then diluted from 1:2 to 1:128 for BoAHV1 and BRSV and 1:8 to 1:512 for BVDV and BPIV3. For the VN test, the virus prototype strains used were Los Angeles (BoAHV1), NADL (BVDV), SF4/32 (BPIV3), and A51908 (BRSV) adapted in MDBK (Madin-Darby bovine kidney) cell culture. The titers of the viral strains used in the VN test were standardized in 100 TCID_50%_. Virus and serum dilutions were incubated for 1 h at 37 °C in a 5% CO_2_ atmosphere. Afterward, 50 μL of 3 × 10^5^ MDBK cells/mL was added. The interpretation of the results was performed after 72 h of incubation at 37 °C. The neutralizing titer of each serum sample was considered the reciprocal of the highest dilution able to neutralize viral replication. Titers ≥ 2 were considered positive for BoAHV1 and BRSV and ≥8 for BVDV and BPIV3.

### 2.3. Statistical Analysis

The seropositivity frequency of viral agents was compared with the type of management and herd size by a 2 × 2 proportion test adjusted by Bonferroni correction [31]. For descriptive analysis, the data were expressed in total number and percentage (%). All statistical analyses were performed using the Minitab^®^ statistical program version 18.1.

## 3. Results

BPIV3-VN antibodies were present in 96.8% (481/497) of the serum samples analyzed, indicating a high frequency of infection in this study. The seropositive rates for BoAHV1, BVDV, and BRSV were 71.4% (355/497), 56.3% (280/497), and 63.4% (315/497), respectively.

Only 3 (0.6%) serum samples from different herds and counties tested negative for all 4 viruses evaluated. In the other 494 serum samples, VN antibodies were identified characterizing the infection by BoAHV1, BVDV, BPIV3, and BRSV as single (*n* = 80; 16.2%), double (*n* = 109; 22.1%), and triple (*n* = 87; 17.6%); however, the presence of VN antibodies against the 4 viruses was the most frequent (*n* = 218; 44.1%) infection type. Table 1 shows the distribution of VN antibodies for BoAHV1, BVDV, BPIV3, and BRSV according to the infection type.

The herd was considered seropositive for the viruses evaluated when VN antibodies against BoAHV1, BVDV, BPIV3, or BRSV were detected in at least one serum sample. Of the 39 dairy herds evaluated, 12 (30.8%) were negative for at least 1 virus included in the analysis. Moreover, four herds were negative for BoAHV1 and one for BVDV. A total of 7 herds were simultaneously negative for more than one virus (BoAHV1 and BVDV, *n* = 1; BoAHV1 and BRSV, *n* = 1; BVDV and BRSV, *n* = 3; and BoAHV1, BVDV, and BRSV, *n* = 2). All 39 (100%) herds had cows with VN antibody titers against BPIV3.

The highest rates of BPIV3 and BRSV seropositivity occurred in herds with semi-intensive management. In contrast, the highest frequencies of BoAHV1-seropositive cows were identified in herds under intensive management (Table 2). The rate of seropositive cows for BoAHV1 presented a statistical difference among the three types of management, whereas the percentage of cows with VN anti-BVDV antibodies did not show any difference. The percentage of seropositivity per herd evaluated and distributed according to the management type is shown in Table S1.

The 39 evaluated herds, according to the number of cows, were subdivided into 4 categories (≤100; 101–250; 251–500, and >500 cows). The lowest frequencies of seropositivity for BoAHV1, BVDV, and BRSV were observed in herds with a maximum of 100 cows. The rate of cows seropositive for BPIV3 was above 90%, regardless of herd size (Table 3). Herds with >500 cows presented a higher proportion of animals seropositive for BVDV and BRSV than smaller herds.

## 4. Discussion

BRD is a significant cause of increased morbidity and mortality in cattle herds and may affect the economy of the livestock sector, particularly the milk production chain [4]. In dairy cattle herds, BRD can occur in both adult animals and suckling as well as newly weaned calves [7,32]. Understanding the epidemiological profile, particularly of viral infections in unvaccinated cows, allows for the assessment of the risk of respiratory infections in calves.

The highest rate (96.8%) of seropositive cows was for BPIV3 and the lowest (56.3%) was for BVDV. The seropositivity rates for BoAHV1 (71.4%) and BRSV (63.4%) were intermediate but higher than 50% of the evaluated samples. In Brazil, Hoppe et al. [15] conducted a study similar to the present survey. The authors analyzed 1,243 sera samples from 26 dairy cattle herds of São Paulo State, Brazil, using the VN test. The present study and the one conducted by Hoppe et al. [15] differed in two points. The authors did not evaluate antibodies to BPIV3, and the sampling included 28.3% (*n* = 352) of calf serum samples. The frequency rates of VN antibodies to BoAHV1, BVDV, and BRSV were 52.3%, 26.6%, and 79.5%, respectively, and only small dairy cattle herds consisting of 6–150 animals were evaluated, with a predominance of herd < 100 animals.

Of the 39 dairy herds included in the study, 35 (89.7%) had cows with high VN antibody titers against BoAHV1 (≥64) or BVDV (≥128). Because only unvaccinated herds were selected, the high antibody titers identified in the VN test indicated recent viral circulation in the herds. Among the 35 herds with cows with high VN antibody titers, 23 (65.7%) also had seronegative cows. Two situations may explain the presence of seronegative animals in the seropositive group. First, the seronegative cows may have been infected. However, in the absence of viral (BoAHV1 or BVDV) circulation in the herd, antibody titers dropped to a level that the VN test could not identify. Second, the cows may not have been infected. In this case, in the presence of BoAHV1, BVDV, or both, confirmed by the identification of cows with high antibody titers, seronegative pregnant cows may develop reproductive problems. Embryonic or fetal mortality may occur depending on the gestational period.

Comparing the viruses separately, several studies described serology in adult and young dairy animals by VN, testing only one of the four viruses or more than one (Table 4). Comparative analyses of the frequencies of seropositive animals for BoAHV1, BVDV, BRSV, and BPIV3 between studies carried out in Brazil, and even in other continents, is a great challenge. Many variables exist and include aspects related to the epidemiology of infections, sampling (*n*), animal age, herd size, type of management (intensive, semi-intensive, or extensive), herd purpose (beef or dairy), production cycle (complete or segmented), herd type (open or closed), active search and elimination of animals persistently infected with BVDV, internal and external biosecurity standards, presence or absence of an immunoprophylactic program for the evaluated viruses, and the serological diagnostic method (VN × ELISA), which make comparisons difficult. Table 4 presents the seroconversion rates for the main respiratory viruses identified in this study and other seroepidemiological surveys conducted in Brazil.

In this study, except for BPIV3, for which more than 95% of the cows were seropositive regardless of herd size, the epidemiological profile of infections was distinct for the other 3 respiratory viruses. The rates of seropositive cows infected with BRSV, BVDV, and BoAHV1 increased considerably as the number of cows in the herds increased (Table 3). In summary, in the dairy cattle herds evaluated in this study, which constituted up to 100 cows, the risk of BRD in adult animals and in heifer calves, due to the absence of passive immunity, was much higher than that in the other size categories of cattle herds evaluated. In addition, in these smaller herds, the risk of reproductive problems due to infections caused by BoAHV1 and BVDV was also high, as the percentage of seronegative cows was much higher than that of the average of the total samples.

The acquisition of females for replacement, which characterizes the herd as open, could be a risk factor for BoAHV1 or BVDV infections. These two viruses were chosen because the viral latency in the case of BoAHV1 [41] and the presence of animals persistently infected with BVDV [42] could be a source of infection for unvaccinated herds. However, compared to herds considered closed, the samples (herds and sera) obtained from open herds did not support the statistical analysis. Of the only 7 open herds, 4 were included in the category of herds with fewer than 100 animals, and 3 in the category with 101 to 250 animals. As a result, the number of serum samples collected from these herds was small, representing only 8.6% (43/497) of the total samples analyzed.

In summary, smaller dairy cattle herds with extensive management have a lower circulation of BoAHV1 and BVDV, consequently decreasing the risk of infection. However, intensive management of large herds increases the risk of infection with these viruses, causing respiratory and reproductive problems in dairy cattle herds. Two biological characteristics of these viruses facilitate the spread of the infection in herds with more cows and intensive management.

## 5. Conclusions

This study showed the presence of VN antibody titers for the four main viruses (BoAHV1, BVDV, BPIV3, and BRSV) involved in episodes or outbreaks of BRD in adult animals and suckling and weaned calves of unvaccinated high-production dairy cattle herds from the central-eastern mesoregion of Parana State. Herds with the highest number of cows and confined management had the highest rates of seropositivity for the viruses evaluated. The lack of vaccination for viral agents commonly associated with respiratory diseases in the herds analyzed and the rate of seropositive cows indicate natural exposure to the viral agents. BPIV3 was the virus with the highest seropositivity rate in animals from the evaluated region.

Sanitary risk reduction in these herds should consider the adoption of appropriate health management as an internal and external biosecurity rule in association with a well-designed immunoprophylactic program. These actions aim to reduce viral circulation among different animal categories as well as the frequency and intensity of episodes or outbreaks of BRD in high-production dairy cattle herds.

## Figures and Tables

**Table 1:** Presence of virus neutralization antibodies to BoAHV1, BVDV, BPIV3, and BRSV in cows, distributed according to the type (single or multiple) of infection.

Infection Type	Virus ^(^*^)^	Number of Cows (%)
Single	BoAHV1	8 (1.6)
	BPIV3	71 (14.3)
	BRSV	1 (0.2)
Double	BVDV + BPIV3	2 (0.4)
	BVDV + BRSV	1 (0.2)
	BPIV3 + BRSV	15 (3.0)
	BoAHV1 + BPIV3	91 (18.3)
Triple	BVDV + BPIV3 + BRSV	49 (9.9)
	BoAHV1 + BPIV3 + BRSV	28 (5.6)
	BoAHV1 + BVDV + BRSV	3 (0.6)
	BoAHV1 + BVDV + BPIV3	7 (1.4)
Quadruple	BoAHV1 + BVDV + BPIV3 + BRSV	218 (44.1)

* BoAHV1 (bovine alphaherpesvirus 1); BVDV (bovine viral diarrhea virus); BPIV3 (bovine parainfluenza virus 3); and BRSV (bovine respiratory syncytial virus).

**Table 2:** BoAHV1, BVDV, BPIV3, and BRSV-seropositive lactating cows, distributed according to the management type of dairy cattle herds in the state of Paraná, Brazil.

Management Type	No. Cows	Number of Cows (%)			
		BoAHV1 (M ± SE)	BVDV (M ± SE)	BPIV3 (M ± SE)	BRSV (M ± SE)
Extensive	44	5 (11.4) ^c^ (6.25 ± 6.25)	22 (50.0) ^a^ (35.8 ± 22.5)	42 (95.4) ^ab^ (97.43 ± 1.48)	22 (50.0) ^b^ (35.8 ± 22.5)
Semi-intensive	131	79 (60.3) ^b^ (58.7 ± 10.4)	77 (58.8) ^a^ (52.9 ± 10.5)	130 (99.2) ^a^ (97.78 ± 2.22)	96 (73.3) ^a^ (78.33 ± 8.29)
Intensive	312	268 (85.9) ^a^ (86.02 ± 5.68)	178 (57.1) ^a^ (56.76 ± 9.19)	299 (95.8) ^b^ (95.68 ± 2.02)	189 (60.6) ^b^ (59.56 ± 9.34)

BoAHV1 (bovine alphaherpesvirus 1); BVDV (bovine viral diarrhea virus); BPIV3 (bovine parainfluenza virus 3); and BRSV (bovine respiratory syncytial virus). ^a–c^ in the same column indicates a statistical difference (*p* ≤ 0.047/Bonferroni correction) among management types.

**Table 3:** Distribution of serum samples from positive cows for the main respiratory viruses, according to the size of the dairy herd, Paraná, Brazil.

Numbers			Positive Samples (%) *			
Cows	Herds	Samples	BoAHV1	BVDV	BPIV3	BRSV
≤100	7	25	4 (16.0) ^c^	5 (20.0) ^c^	24 (96.0) ^ab^	13 (52.0) ^b^
101–250	15	205	131 (63.9) ^b^	106 (51.7) ^b^	203 (99.0) ^a^	121 (59.0) ^b^
251–500	10	142	113 (79.6) ^a^	81 (57.0) ^b^	135 (95.1) ^b^	88 (62.0) ^b^
>500	7	125	107 (85.6) ^a^	88 (70.4) ^a^	119 (95.2) ^ab^	93 (74.4) ^a^

^(^*^)^ BoAHV1 (bovine alphaherpesvirus 1); BVDV (bovine viral diarrhea virus); BPIV3 (bovine parainfluenza virus 3); and BRSV (bovine respiratory syncytial virus). ^a–c^ in the same column indicates a statistical difference (*p* ≤ 0.047/Bonferroni correction) among management types.

**Table 4:** Seroconversion rates for the four main respiratory viruses identified in this study and other serological surveys conducted in dairy cattle herds in Brazil.

Age (mos)	Samples (*n*)	Percentage of Positive Samples				Reference
		BoAHV1	BVDV	BRSV	BPIV3	
>24	497	71.4	56.3	63.4	96.8	This study
>30	977	41.9	nt	nt	nt	[33]
>30	204	32.8	29.4	nt	nt	[34]
>24	180	31.1	nt	nt	nt	[35]
<12 and >12	1243	52.3	26.6	62.8	nt	[15]
>12	317	nt	17.03	nt	nt	[36]
<12	145	31.7	24.4	37.4	nt	[21]
<12	44	54.5	72.3	65.9	67.3	[37]
uninformed	200	57.7	57.7	nt	nt	[38]
uninformed	600	nt	nt	61.3	nt	[29]
uninformed	1161	66.7	nt	nt	nt	[39]
uninformed	264	62.5	45.1	nt	nt	[40]

BoAHV1 (bovine alphaherpesvirus 1); BVDV (bovine viral diarrhea virus); BPIV3 (bovine parainfluenza virus 3); and BRSV (bovine respiratory syncytial virus); nt (not tested).

## Data Availability

Data are contained within the article and Supplementary Materials.

## Supplementary Materials

The following supporting information can be downloaded at https://www.mdpi.com/article/10.3390/ani14091256/s1, Table S1: Seropositive lactating cows per herd for BoAHV1, BVDV, BPIV3, and BRSV virus neutralizing antibodies distributed according to the type of management (A, B, and C) in dairy herds in Paraná. Brazil.
