# Supplementary material for: Serological Profile for Major Respiratory Viruses in Unvaccinated Cows from High-Yielding Dairy Herds
Source: Animals (Basel). 2024 Apr 23;14(9):1256. doi: 10.3390/ani14091256 (PMC11083270; doi:10.3390/ani14091256)
Supplement: Supplementary file 1 [file animals-14-01256-s001.zip › animals-2924866-supplementary.pdf]

Table S1. Seropositive lactating cows per herd for BoAHV1, BVDV, BPIV3, and BRSV virus neutralizing antibodies distributed according to the type of management (A, B, and C) in dairy herds in Paraná. Brazil.

Management type	Herd	Samples (n)	% seropositive animals			
			BoAHV1	BVDV	BPIV3	BRSV
Extensive (A)	A1	19	0 (0/19)	100 (19/19)	94.74 (18/19)	100 (19/19)
	A2	2	0 (0/2)	0 (0/2)	0 (2/2)	0 (0/2)
	A3	3	0 (0/3)	33.33 (1/3)	100 (3/3)	33.33 (1/3)
	A4	20	25 (5/20)	10 (2/20)	95 (19/20)	10 (2/20)
Subtotal		44	11.4 (5/44)	50 (22/44)	95.4 (42/44)	50 (22/44)
Semi-intensive (B)	B1	20	20 (4/20)	100 (20/20)	100 (20/20)	100 (20/20)
	B2	20	35 (7/20)	30 (6/20)	100 (20/20)	30 (6/20)
	B3	8	100 (8/8)	75 (6/8)	100 (8/8)	62.5 (5/8)
	B4	8	100 (8/8)	75 (6/8)	100 (8/8)	100 (8/8)
	B5	8	100 (8/8)	62.5 (5/8)	100 (8/8)	87.5 (7/8)
	B6	8	37.5 (3/8)	0 (0/8)	100 (8/8)	50 (4/8)
	B7	8	12.5 (1/8)	0 (0/8)	100 (8/8)	0 (0/8)
	B8	8	100 (8/8)	100 (8/8)	100 (8/8)	100 (8/8)
	B9	8	75 (6/8)	37.5 (3/8)	100 (8/8)	50 (4/8)
	B10	20	100 (20/20)	80 (16/20)	100 (20/20)	95 (19/20)
	B11	3	66.67 (2/3)	33.33 (1/3)	100 (3/3)	100 (3/3)
	B12	3	100 (3/3)	100 (3/3)	100 (3/3)	100 (3/3)
	B13	3	33.33 (1/3)	100 (3/3)	100 (3/3)	100 (3/3)
	B14	3	0 (0/3)	0 (0/3)	100 (3/3)	100 (3/3)
	B15	3	0 (0/3)	0 (0/3)	66.67 (2/3)	100 (3/3)
Subtotal		131	60.3 (79/131)	58.8 (77/131)	99.2 (130/131)	73.3 (96/131)
Intensive (C)	C1	11	81.82 (9/11)	0 (0/11)	63.64 (7/11)	0 (0/11)
	C2	19	100 (19/19)	94.74 (18/19)	94.74 (18/19)	100 (19/19)
	C3	17	100 (17/17)	94.12 (16/17)	100 (17/17)	100 (17/17)
	C4	19	100 (19/19)	78.95 (15/19)	100 (19/19)	94.74 (18/19)
	C5	19	94.74 (18/19)	57.89 (11/19)	100 (19/19)	73.68 (14/19)
	C6	18	94.44 (17/18)	72.22 (13/18)	100 (18/18)	72.22 (13/18)
	C7	19	100 (19/19)	94.74 (18/19)	94.74 (18/19)	94.74 (18/19)
	C8	18	83.33 (15/18)	88.89 (16/18)	100 (18/18)	88.89 (16/18)
	C9	19	100 (19/19)	100 (19/19)	100 (19/19)	100 (19/19)
	C10	19	100 (19/19)	31.58 (6/19)	100 (19/19)	52.63 (10/19)
	C11	20	70 (14/20)	10 (2/20)	90 (18/20)	10 (2/20)
	C12	20	100 (20/20)	0 (0/20)	95 (19/20)	0 (0/20)
	C13	20	60 (12/20)	10 (2/20)	100 (20/20)	15 (3/20)
	C14	8	62.5 (5/8)	75 (6/8)	100 (8/8)	75 (6/8)
	C15	8	87.5 7/8)	0 (0/8)	100 (8/8)	0 (0/8)
	C16	8	100 (8/8)	75 (6/8)	100 (8/8)	100 (8/8)
	C17	20	100 (20/20)	90 (18/20)	85 (17/20)	95 (19/20)
	C18	19	0 (0/19)	5.26 (1/19)	94.74 (18/19)	5.26 (1/19)
	C19	11	100 (11/11)	100 (11/11)	100 (11/11)	54.55 (6/11)
Subtotal		312	85.9 (268/312)	57.1 (178/312)	95.8 (299/312)	60.6 (189/312)
**Total**		**487**	**72.3 (352/487)**	**56.9 (277/487)**	**96.7 (471/487)**	**63.0 (307/487)**

BoAHV1 (Bovine alphaherpesvirus 1); BVDV (Bovine viral diarrhea virus); BPIV3 (Bovine parainfluenzavirus 3); BRSV (Bovine respiratory syncytial virus).
